# Parallel and serial task processing in the PRP paradigm: a drift–diffusion model approach

**DOI:** 10.1007/s00426-020-01337-w

**Published:** 2020-04-25

**Authors:** André Mattes, Felice Tavera, Anja Ophey, Mandy Roheger, Robert Gaschler, Hilde Haider

**Affiliations:** 1 Department of Psychology, University of Cologne, Richard-Strauss-Straße 2, 50931 Cologne, Germany; 2 Department of Medical Psychology, Neuropsychology and Gender Studies and Center for Neuropsychological Diagnostics and Intervention (CeNDI), Medical Faculty, University Hospital Cologne, Kerpener Str. 68, 50937 Cologne, Germany; 3 Department of Psychology, FernUniversität in Hagen, Universitätsstraße 33, 58084 Hagen, Germany

## Abstract

Even after a long time of research on dual-tasking, the question whether the two tasks are always processed serially (response selection bottleneck models, RSB) or also in parallel (capacity-sharing models) is still going on. The first models postulate that the central processing stages of two tasks cannot overlap, producing a central processing bottleneck in Task 2. The second class of models posits that cognitive resources are shared between the central processing stages of two tasks, allowing for parallel processing. In a series of three experiments, we aimed at inducing parallel vs. serial processing by manipulating the relative frequency of short vs. long SOAs (Experiments 1 and 2) and including no-go trials in Task 2 (Experiment 3). Beyond the conventional response time (RT) analyses, we employed drift–diffusion model analyses to differentiate between parallel and serial processing. Even though our findings were rather consistent across the three experiments, they neither support unambiguously the assumptions derived from the RSB model nor those derived from capacity-sharing models. SOA frequency might lead to an adaptation to frequent time patterns. Overall, our diffusion model results and mean RTs seem to be better explained by participant’s time expectancies.

## Introduction

Research in the field of dual-tasking so far showed that performing more than one task concurrently produces costs, even with quite simple tasks (Koch, Poljac, Müller & Kiesel, 2018). These dual-task costs have been extensively investigated within the Psychological Refractory Period (PRP) paradigm (Telford, 1931). Here, participants are instructed to concurrently conduct two stimulus–response tasks that are separated by a variable Stimulus-Onset Asynchrony (SOA). Whereas a manipulation of the SOA has only minor effects on primary task processing, response times (RT) of the secondary task increase with shorter SOAs. Based on the locus-of-slack method (Schweickert, 1978), Pashler (1994b) explored why the secondary response is postponed. By systematically manipulating each of three processing stages (i.e., stimulus encoding, response selection, and response production), he concluded that most likely the crucial limitation (or bottleneck) in dual-task performance is located in the response selection stage. In the subsequent Response Selection Bottleneck (RSB) model, Pashler (1994a) suggested that only one single response can be selected at a time. Therefore, response selection for the secondary task can start only after the response selection process of the primary task has finished. In this framework, task processing in a dual-task paradigm is thought to be strictly serial (Pashler, 1998).

However, since Pashler’s seminal proposal of the RSB model, at least three classes of findings pose a challenge to the RSB model, because they might reflect parallel task processing. First, compelling evidence suggests that perfect time-sharing after intensive dual-task training is possible (Allport, Antonis, & Reynolds, 1972; Halvorson, Ebner & Hazeltine, 2013; Halvorson & Hazeltine, 2015; Israel & Cohen, 2011; Schumacher et al., 2001). Second, it has been demonstrated that an incompatibility between the primary and secondary tasks’ responses does not only prolong the RT of the secondary task but also that of the primary task (Hommel, 1998), a phenomenon known as the backward crosstalk effect (BCE; Hommel, 1998; Janczyk, Pfister, Hommel, & Kunde, 2014; Logan & Schulkind, 2000; Miller, 2006). Third, several findings suggest that participants can strategically switch between mostly serial and mostly parallel task processing modes (Fischer, Gottschalk, & Dreisbach, 2014; Fischer & Dreisbach, 2015; Fischer & Plessow, 2015; Lehle & Hübner, 2009; Miller, Ulrich & Rolke, 2009). While the first two classes of findings are, at least in principle, reconcilable with the framework of the RSB model, the third class of findings is more difficult to explain (but see Logan & Gordon, 2001).

With regard to the findings of no dual-task costs, Schubert et al. (2017) propose that practice might improve the inter-task coordination, thereby optimizing processing in the bottleneck. This is supported by results that show residual dual-task costs even after extensive training of eight sessions (Liepelt, Fischer, Frensch, & Schubert, 2011). According to this latent bottleneck perspective (Ruthruff, Johnston, Van Selst, Whitsell, & Remington, 2003), processing optimized for the bottleneck rather than a parallel processing mode leads to the reduced dual-task costs (e.g., Strobach & Schubert, 2017).

To integrate the second class of findings that demonstrate a BCE into the framework of the RSB model, Hommel (1998) argued that, based on the ideomotor principle (e.g., Prinz, 1984; Stock & Stock, 2004), merely perceiving the secondary stimulus automatically activates features of the secondary response (Hommel & Eglau, 2002; Hommel, 1998; Janczyk et al., 2014; Röttger & Haider, 2017). Crucially, the activation of these response features can proceed in parallel to other processing stages of Task 1, thereby causing crosstalk between the tasks. Distinguishing between response feature activation and response selection allows to account for a BCE despite the assumption of a structural bottleneck within the RSB model (Lien & Proctor, 2000; Paelecke & Kunde, 2007, but see Thomson, Danis, & Watter, 2015; Janczyk, Renas, & Durst, 2018). Thus, although the two classes of findings might point to parallel processing, they are consistent with an extended RSB model (Hommel, 1998; Lien & Procter, 2000; Schubert, Fischer, & Stelzel, 2008).

Yet, the third class of findings that assumes an ability to adaptively switch between a mostly parallel and a mostly serial processing mode (Fischer et al., 2014; Fischer & Dreisbach, 2015; Fischer & Plessow, 2015; Lehle & Hübner, 2009; Miller et al., 2009), is more difficult to explain within the framework of the RSB model since it requires the assumption of higher-order control processes for active task scheduling or coordination (cf. Logan & Gordon, 2001). For instance, Fischer et al. (2014) confronted participants with either high or low conflicting task contexts by manipulating response compatibility between the primary and the secondary task. In contexts with high response conflict, the RT differences between compatible and incompatible tasks were reduced. The authors interpret these findings in favor of more task shielding and, thus, a switch to a mostly serial processing strategy.

In a similar vein, Lehle and Hübner (2009) manipulated participants’ performance via instruction. They used a version of the Eriksen flanker task in which participants had to respond to the target (primary task) and to the flankers (secondary task). They instructed their participants to conduct the two tasks in either a parallel or serial processing mode. The results showed that performance was more prone to task interference under parallel than under serial task processing instructions, even though participants showed a bias towards parallel processing when no specific instruction was presented. In a follow-up study, Lehle, Steinhauser, and Hübner (2009) could demonstrate that mental effort, reflected by heart rate and electrodermal activity, was higher under serial processing compared to parallel processing. The authors concluded that participants preferred a processing mode that required less mental effort and, thus, by default, adopted a moderate parallel processing strategy.

Overall, these findings suggest an adaptive modulation of processing strategies. Such a modulation can hardly be explained by the RSB model, as, according to its premises, the tasks should always be processed in a strictly serial processing mode. However, such findings can easily be accommodated by capacity-sharing models (Navon & Miller, 2002; Tombu & Jolicoeur, 2003) or by cognitive control models (cf. Logan & Gordon, 2001).

Capacity-sharing and cognitive control models both propose that it is not a structural limitation but the limited central capacity or conflicts (crosstalk) between the two tasks which narrows the possibility of concurrently conducting two tasks. If the processing of the two tasks overlaps in time, the limited capacity needs to be shared between them leading to parallel response selection, but—due to the shared limited capacity—also to dual-task costs and the PRP effect. Since it is additionally assumed that people can strategically distribute their limited capacities between the two tasks (e.g., Logan & Gordon, 2009; Tombu & Jolicoeur, 2003), the scope of this second class of theories is broader and more flexible than that of structural bottleneck theories. These models cannot only account for findings suggesting parallel processing, such as dual-tasking without any costs under specific conditions (Allport et al., 1972; Halvorson et al., 2013; Halvorson & Hazeltine, 2015; Israel & Cohen, 2011; Schumacher et al., 2001) or the BCE (Janczyk et al., 2014; Logan & Schulkind, 2000). Additionally, they can account for findings suggesting serial processing (Miller et al., 2009) or for those showing that participants can adaptively switch between parallel and serial processing modes (Fischer et al., 2014; Fischer & Dreisbach, 2015; Lehle & Hübner, 2009).

However, at least a few recent findings challenge the assumption of parallel task processing (Marti, Sigman, & Dehaene, 2012; Maslovat et al., 2013; Ruthruff, Johnston, & Remington, 2009). For instance, Maslovat et al. (2013) tested whether the second response in a single-choice dual-task paradigm is already prepared, while participants respond to the first task. They used a startle acoustic stimulus (SAS) that was intended to trigger more or less involuntarily an already prepared response. They found that with presenting the SAS at different SOAs, the secondary response was accelerated compared to a control condition without SAS. Yet, this effect was additive to the PRP effect, but, according to the second class of models, this effect should have been stronger for short than for long SOAs.

To summarize, the debate about whether the RSB or models allowing also for parallel processing are better suited to account for dual-task performance continues (e.g., Mittelstaedt & Miller, 2017). On the one hand, capacity-sharing and cognitive control models can more flexibly account for the current findings in multitasking than the RSB model. On the other hand, empirical findings unambiguously favoring either the one or the other class of models are still rare.

The goal of the current experiments is to contribute to this debate by focusing in particular on the question of parallel versus serial task processing. Building on a study of Miller et al. (2009), we first report three experiments aimed at establishing parallel versus serial processing in a dual-task setting. In a second step, we explore assumptions of parallel and serial task processing modes by means of the drift–diffusion model (Ratcliff & Rouder, 1998). The drift–diffusion model is a computational model which decomposes the single trial RTs into different underlying components. These components, reflected in the model’s parameters, can be linked to cognitive processes (e.g., Janczyk & Lerche, 2019; Voss, Voss, & Lerche, 2015). The analysis of these components could help to distinguish between parallel and serial processing strategies.

## Overview of the current study

The central assumption put forward by Miller et al. (2009) is that the selection of either a parallel or a serial processing mode in dual-tasking is driven by the goal to minimize the total reaction time (TRT), which is the time needed to conduct both tasks. We see this TRT framework as instantiation of capacity-sharing accounts. Based on mathematical simulations, Miller et al. (2009) demonstrate that serial processing is usually more efficient than parallel processing. The only situation where parallel processing results in shorter TRTs is when short SOAs are frequently present within dual-task blocks.

To provide empirical evidence for this assumption, the authors ran three experiments in which they compared a block with frequently short SOAs against another block with frequently long SOAs. With frequently short SOAs, the RTs to the first task were slower than with frequently long SOAs. Concurrently, the secondary tasks’ RTs at short SOAs were faster in the frequently short SOA than in the frequently long SOA blocks. This is exactly what one would expect to find under the assumption that parallel processing is adopted when short SOAs are frequent: The parallel response selection in the frequently short SOA blocks reduces the speed of responding to the primary task and concurrently speeds up the processing in the secondary task given short SOAs, leading to flatter slopes. Thus, the findings are in line with the assumption that optimizing the TRT is obtained by the use of a mostly parallel versus a mostly serial processing mode within the PRP paradigm.

Yet, one finding of Miller et al. (2009) might be at odds with the assumption of mostly parallel task processing in the frequently short condition. In Task 1, the larger the RT differences between the frequently short and the frequently long conditions, the longer the SOAs (Experiments 1 and 2). If participants in the frequently short condition had shared their capacities between the two tasks, the reverse pattern should have been found as the need for capacity sharing should be larger when the SOAs are short.

In the current study, we build on the experimental strategy of Miller et al. (2009). In all experiments, participants received either 80% short SOAs (short SOAs frequent (SF) condition) or 80% long SOAs (long SOAs frequent (LF) condition).

In Experiment 1, the SOAs were set to either 100 ms, 300 ms, or 800 ms (in contrast to 16, 133, 500, and 1000 ms in Miller et al. 2009). In Experiment 2, we prolonged the SOAs to 300 ms, 500 ms, or 1000 ms. According to Miller et al. (2009), the goal to minimize the TRT triggers participants to prefer a mostly parallel or a mostly serial processing strategy. If this was true, the longer SOAs in Experiment 2 should increase the likelihood of serial processing irrespectively of whether short or long SOAs are frequent. Thus, the RT differences between the two SOA conditions should decrease in Experiment 2. In Experiment 3, we used the SOAs of Experiment 1, but enhanced the prioritization of the primary task by providing a few trials in which participants had to respond only to the primary task. As already shown by Mittelstädt and Miller (2017), such Task 2 no-go trials increase the likelihood of a serial processing strategy. Therefore, we assumed that this manipulation should also decrease the RT differences between the two SOA conditions.

To summarize, we expected to find indicators for mostly parallel processing in the SF condition and for mostly serial processing in the LF condition of Experiment 1. The additional manipulations in Experiments 2 and 3 should reduce these differences between the two SOA conditions. In a second step, we modeled the data with a diffusion model approach for further insights in the performance differences between the two experimental conditions.

## Drift–diffusion model

**Fig. 1:**
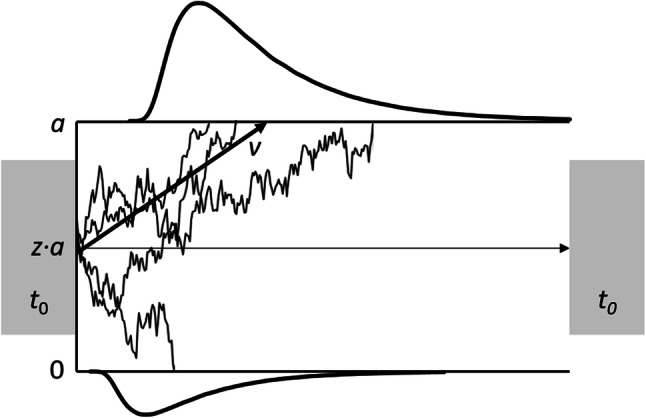
Exemplary illustration of the Drift–Diffusion Model. *za* indicates the starting point of the evidence accumulation process progressing with drift rate *v* until it reaches the threshold *a* or 0. The non-decision time *t*_0_ includes both the stimulus encoding time and the response execution process

Drift–diffusion models are computational methods describing the RT distributions in binary decision tasks (Fig. 1; Ratcliff & McKoon, 2008). The crucial assumption is that a response to a stimulus can be decomposed into four different components (Voss et al., 2015). The decision process for the one or the other response relies on the evidence accumulation process, represented as the drift rate *v*. Evidence accumulation continues until it reaches one of the two thresholds (boundary separation *a*), indicating that an overt response is initiated. The evidence accumulation process can start either at a neutral starting point (*z* = 0.5 *a*) or at a starting point closer to one of the two thresholds (*z* < 0.5 *a* or *z* > 0.5 *a*). In the latter case, the evidence accumulation process would be biased towards one of the two responses. In addition, all processes outside the decision process (e.g., stimulus encoding or motor processes) are subsumed in the non-decision time *t*_0_. In numerous empirical studies, these parameters have been linked to distinct cognitive processes (Durst & Janczyk, 2019; Janczyk & Lerche, 2019; Lerche & Voss, 2017; Naefgen, Dambacher & Janczyk, 2018; Ratcliff & Rouder, 1998; Voss, Nagler & Lerche, 2013).

**Fig. 2:**
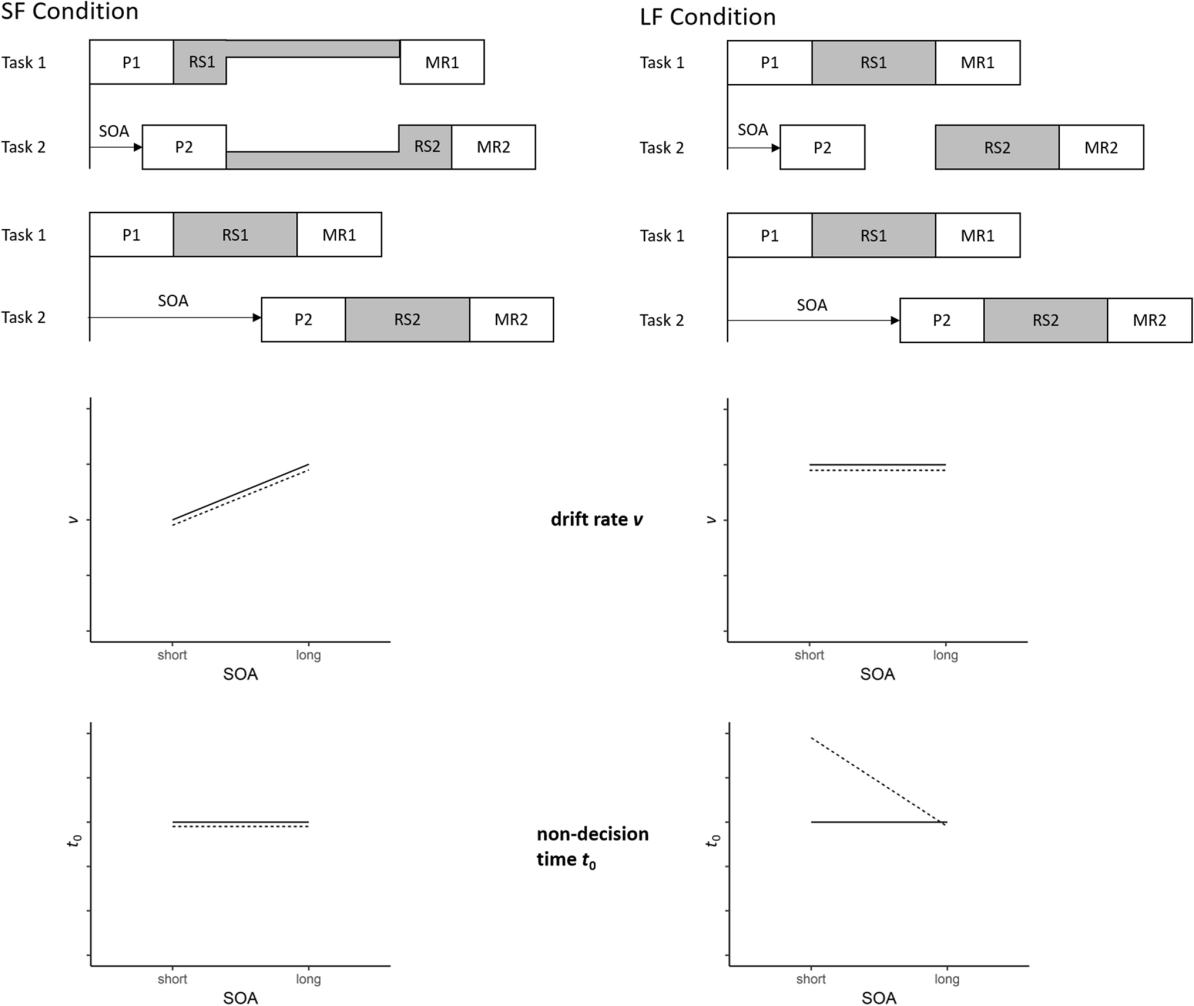
Illustration of the predicted SF (left) and LF (right) processing mode and resulting predictions regarding drift rate *v* and non-decision time *t*_0_. P1/P2, stimulus perception in Task 1/Task 2; RS1/RS2, response selection in Task 1/Task 2; MR1/MR2, motor response in Task 1/Task 2. Solid line, predictions for Task 1; dotted line: predictions for Task 2

With regard to dual-task experiments, only few researchers have already applied the drift–diffusion model (e.g., Durst & Janczyk, 2019; Kamienkowski, Pashler, Dehaene & Sigman, 2011). Nonetheless, for the research question at hand, this approach is promising. The drift–diffusion model makes precise predictions about the parameter configurations for parallel and serial processing and how these parameters should differ (see also Fig. 2).

The first prediction concerns the drift rate *v*: Participants in the SF condition should be more likely to process the two tasks in parallel. This means that for short SOAs, the central processing stages of Task 1 and Task 2 overlap. Due to this overlap, the central resources need to be shared, decreasing the speed of evidence accumulation reflected by the drift rate *v*. For long SOAs, however, this time overlap would be smaller and consequently the need to share central resources would be reduced. Accordingly, in the SF condition, the drift rate should be lower for short SOAs than for long SOAs. By contrast, participants in the LF condition should prefer a serial processing mode. Consequently, there should not be an overlap of the central processing stages of Task 1 and Task 2 neither for long nor for short SOAs. Therefore, the length of the given SOAs should not affect the drift rate. In addition, with short SOAs, the drift rate should be larger in the LF than in the SF condition.

The second prediction concerns the non-decision time *t*_0_: In the diffusion model, the non-decision time comprises all processes which are not related to the decision itself. When in serial processing, the central processing stage of Task 2 is postponed because it cannot overlap with the central processing stage of Task 1, this should be mapped onto the non-decision time of Task 2, given that no decisional processes occur during this slack time. In the SF condition, participants should be more likely to process the two tasks in parallel. Accordingly, there should not be a postponement of the response selection in Task 2 and the non-decision times in both tasks exclusively comprise stimulus encoding and the motor response. Thus, there should be no difference in the non-decision time in the SF condition neither between Task 1 and Task 2, nor between short and long SOAs. However, if participants process the tasks serially, as is assumed to be the case in the LF condition, the response selection process of Task 2 must be postponed until the response selection process of Task 1 has finished when SOAs are short. This should be reflected in the non-decision time of Task 2, such that the non-decision time for Task 2 should be prolonged when the SOA is short but not when it is long. Figure 2 summarizes these predictions for the two conditions.

## General method

### Stimulus and apparatus

All experiments were run on a 19-inch screen (resolution: 1280 × 1024 pixels), controlled by a standard PC. Responses were made on a German standard QWERTZ keyboard using the keys “a” and “s” for the primary task (the two outer left keys in the third row of the keyboard) and “ö” and “ä” for the secondary task (the two outer right keys in the third row of the keyboard).

In all experiments, each trial consisted of a color discrimination task (Task 1) and an object-identity task (Task 2). In the color discrimination task, participants were asked to indicate the color of the picture of either a banana or a plum by pressing the correspondingly marked yellow (“a” key) or blue key (“s” key) with their middle or index finger of the left hand. The stimuli of the color discrimination task appeared on the left side of a central fixation cross. In the object-identity task, participants saw either a dog or a donut appearing on the right from the fixation cross and responded with a left or right keypress on the “ö” and “ä” keys. The exact stimulus–key mapping was counterbalanced across participants instructing them to press the right or left key for the dog or donut.

### Procedure

Informed consent was obtained from all individual participants included in the study. The experiments began with computer-based instructions. The task was explained through standardized instruction on the screen, and the stimuli together with their assigned keys were introduced. Then, the practice phase started with a single block in which participants practiced each of the two tasks for 20 trials separately. Afterwards, participants received one warm-up dual-task block with 80 trials. They were instructed to respond as quickly and accurately as possible to both tasks, and to give equal priority to both tasks and not to group their responses. The maximum response window was set to one minute. In each trial of this warm-up block, both, the color stimulus (banana vs. plum) and the object-identity stimulus (dog vs. donut), appeared simultaneously on the screen (SOA = 0 ms) and participants had to respond to both. The rationale behind presenting the two tasks concurrently was that we wanted to give parallel task processing the maximal chance to occur. According to Miller et al. (2009), parallel processing is rarely used because in most cases, serial task processing is the more efficient strategy.

The following test phase consisted of a total of 400 dual-task trials divided into five blocks with 80 trials each. They were separated by short self-paced breaks. Each block included three types of different SOA trials (100, 300, and 800 ms in Experiments 1 and 3, and 300, 500, and 1000 ms in Experiment 2). In the SF condition, each block consisted of 80% short SOAs, 10% medium, and 10% long SOAs. Correspondingly, the LF condition contained 80% long SOAs, 10% medium, and 10% short SOAs. SOAs were crossed with the stimulus combinations and the resulting trials were presented in random order.^1^

Each trial in the dual-task blocks began with the presentation of a central fixation cross for 250 ms. Following a blank screen for 250 ms, the first stimulus (banana or plum) appeared on the left side of the fixation cross. After the respective SOA, the second stimulus (dog or donut) appeared on the right side of the fixation cross. When the participants had made the two responses, the screen went black for 1500 ms and the next trial started. If a participant responded incorrectly, an error message appeared on the screen for 500 ms specifying whether the error occurred in the first, the second, or in both tasks.

### Design and analyses

All three experiments consisted of a 2 × 3 mixed factorial design with SOA condition (SF vs. LF) as between-participants variable and SOA (short vs. medium vs. long) as within-participants variable. RTs and error rates served as dependent variables.

Trials with incorrect responses on either task were excluded from RT analysis, and responses faster than 200 ms or slower than 2000 ms were eliminated (Experiment 1, 2.64%; Experiment 2, 2.68%; Experiment 3, 0.68%). In addition, we checked whether excluding trials with short inter-response intervals (IRIs < 100 ms; Miller et al., 2009) was warranted. Since the exclusion of trials with IRIs between 100 and 200 ms did not alter the results, we decided not to exclude these trials. For further analyses, mean RTs and mean error rates as dependent variables were computed separately for each participant and each SOA. Partial *η*^2^ (*η*_*p*_^2^) are reported as effect sizes. If Mauchly’s test of sphericity reached significance, we report Greenhouse–Geisser-corrected *p *values together with the original degrees of freedom and the value of *ε*. The alpha level for all analyses was set to *α* = 0.05.

### Modeling

We used the HDDM Toolbox (Wiecki, Sofer, & Frank, 2013) to fit the diffusion model to the RT data. The toolbox employs a Bayesian approach to estimate the parameters. Modeling results will be reported for all three experiments together after having covered mean RT-based analyses of the experiments individually.

In contrast to the RT analyses, we only included trials with short or long SOAs, leaving out medium SOAs, because we wanted to keep the analyses as easy as possible. We applied the same exclusion criteria as used for the RT analyses. We fitted one diffusion model to the data of Task 1 and one diffusion model to the data of Task 2 independently from each other. Note that we fitted the models to the accuracy coded data, i.e., the lower threshold 0 represented an error and the upper threshold *a* represented a correct response. In each model, we allowed only the drift rate *v* and the non-decision time *t*_0_ to vary between the two conditions (between-subjects factor: SF vs. LF condition) and SOA (within-subjects factor: short vs. long). Since there was no theoretical reason to expect differences in the boundary separation *a* between the conditions, we did not allow this parameter to vary between the conditions. However, the boundary separation parameter was estimated for every participant individually. Furthermore, we did not expect a response bias towards one of the two responses in the experimental task. Consequently, we fixed the starting point *z* at 0.5*a*, indicating that the evidence accumulation process always starts in the middle between both decision boundaries. The variability parameters of the drift rate, non-decision time and starting point were set to 0 and the diffusion constant was set to *s* = 1.

For each model estimation, we drew 6000 samples from the joint posterior distribution of the parameters using the Markov Chain Monte Carlo (MCMC) sampling and discarded the first 1000 samples as the burn-in period. Thus, the posterior distribution of each parameter consisted of 5000 samples. We examined MCMC convergence by visually inspecting the traces and computing the Ȓ statistic (Gelman & Rubin, 1992).

For the statistical analyses, the HDDM Toolbox produces a posterior distribution of the diffusion model parameters. Within a model, these distributions can be directly compared to each other. To test whether a parameter estimate differed substantially between long and short SOAs within a task, we examined the Bayesian posterior probability based on the group-level nodes. We, thus, computed the proportion *P* of the parameter distribution for short SOAs which was larger/smaller than the parameter distribution for long SOAs. If *P* exceeded 0.950 indicating that 95% of one parameter distribution was larger/smaller than the other parameter distribution, we assumed a substantial difference.

Since the parameters for the different tasks are not estimated simultaneously, unlike the parameters within a task, we could not use Bayesian posterior probabilities to compare the parameters. Instead, we aggregate the posterior distributions per participant by computing the means. We then employed frequentist *t *tests and adjusted the *p *value for the number of comparisons on each parameter and in each experiment using the Bonferroni correction. We provide more information on the technical aspects of the parameter estimation procedure in the "Appendix".

## Experiment 1

The first experiment aimed to replicate the qualitative pattern of results of Miller et al. (2009). Instead of using their SOAs of 16 ms, 133 ms, 500 ms, and 1000 ms, the lengths of the SOAs here were either 100 ms, 300 ms, or 800 ms.

### Method

#### Participants

Thirty-eight students (9 men, mean age *M* = 24.08 years; SD = 6.52) of the University of Cologne took part in the experiment for exchange of either course credit or a payment of 3 Euros. 21 participants were randomly assigned to the SF condition and 17 participants to the LF condition. Due to a high error rate (larger than 15% in all blocks), one participant of the LF condition had to be excluded. When including this participant, the results did not change.

### Results and discussion

We first analyzed participants’ mean error rate (see Table 1). A 2 (SOA condition) × 3 (SOA) ANOVA with mean error rates as dependent variable did not reveal any significant effects (all *F*s ≤ 1.34, *p*s ≥ 0.269 and *F* ≤ 2.97, *p* ≥ 0.058 for Task 1 and Task 2, respectively).

**Table 1:** Mean percent error and standard error in brackets for Experiments 1, 2, and 3 as a function of SOA and SOA conditions

Experiment 1			Experiment 2			Experiment 3^a^		
SOA	Mean [SE]		SOA	Mean [SE]		SOA	Mean [SE]	
	SF condition	LF condition		SF condition	LF condition		SF condition	LF condition
Task 1								
100	1.83 [0.69]	2.64 [0.77]	300	1.35 [0.58]	2.88 [0.59]	100	1.28 [0.56]	2.50 [0.52]
300	2.14 [0.89]	3.67 [0.98]	500	1.78 [0.77]	2.50 [0.79]	300	1.45 [0.61]	1.79 [0.58]
800	2.38 [0.62]	3.19 [0.69]	1000	2.14 [0.59]	2.66 [0.60]	800	1.18 [0.47]	2.01 [0.45]
Task 2								
100	5.73 [1.69]	10.71 [1.88]	300	5.89 [1.16]	4.50 [1.19]	100	3.34 [1.01]	3.69 [0.96]
300	5.00 [1.07]	8.09 [1.20]	500	6.79 [1.31]	4.63 [1.34]	300	3.55 [0.96]	4.40 [0.91]
800	4.88 [1.12]	6.34 [1.32]	1000	5.83 [1.02]	4.30 [1.05]	800	3.68 [0.73]	2.43 [0.69]

^a^No-go trials were excluded

**Fig. 3:**
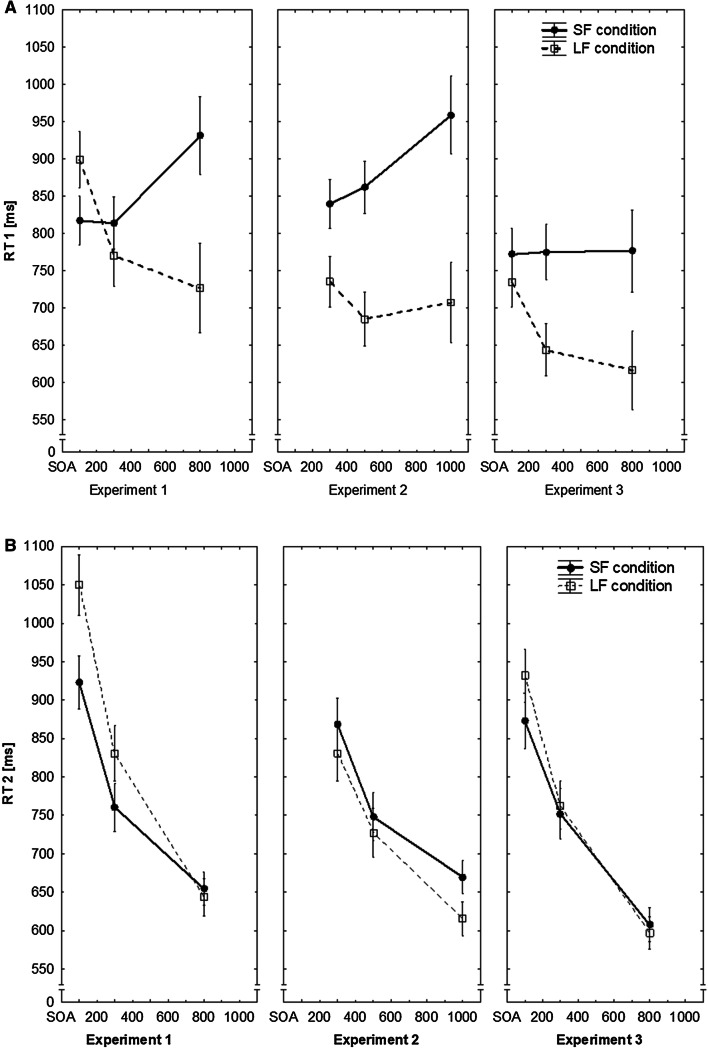
Mean RTs in Tasks 1 (**a**) and 2 (**b**) as a function of SOA and SOA condition in Experiments 1, 2, and 3. Error bars reflect between-participants standard error (SE) of the mean

Figure 3a and b display the mean RTs as a function of SOA for the two experimental conditions in Task 1 and Task 2. The 2 (SOA condition) × 3 (SOA) ANOVA with RT1 as dependent variable revealed a significant main effect of SOA, (*F*(2,70) = 3.71, *p* = 0.029, *η*_*p*_^2^ = 0.10), but no significant main effect of SOA condition, (*F*(1,35) = 0.86, *p* = 0.360, *η*_*p*_^2^ = 0.02). Yet, the SOA condition × SOA interaction reached significance (*F*(2,70) = 17.36, *p* < 0.001, *η*_*p*_^2^ = 0.33; *ε* = 0.598). As can be seen from Fig. 3a, the longer the SOAs were, the slower the participants in the SF condition responded; whereas, participants in the LF condition showed the reversed trend.

Regarding RT2, the analogous 2 × 3 ANOVA yielded a significant main effect for SOA (*F*(2,70) = 436.10, *p* < 0.001, *η*_*p*_^2^ = 0.93; *ε* = 0.941), indicating the expected PRP effect. There was no main effect of SOA condition (*F*(1,35) = 1.81, *p* = 0.186, *η*_*p*_^2^ = 0.05), but the SOA condition × SOA interaction was highly significant (*F*(2,70) = 18.27, *p* < 0.001, *η*_*p*_^2^ = 0.34; *ε* = 0.941). The latter was due to a flatter slope in the SF condition (see Fig. 3b).

Overall, this pattern of results is in line with Miller et al. (2009) who did not find significant main effects for the SOA conditions, neither for RT1 nor RT2, but significant interactions between SOA condition and SOA. Therefore, Experiment 1 replicates the qualitative pattern of the results of Miller et al. (2009) that are interpreted in favor of the assumption of mostly parallel processing in the SF and mostly serial processing in the LF condition.

## Experiment 2

The goal of Experiment 2 was to test whether prolonging the shortest SOA would reduce the likelihood of parallel processing in the SF condition. According to the TRT account (Miller et al., 2009), such a prolongation of the SOAs should decrease the likelihood that the parallel processing strategy is more efficient than a serial one. Consequently, the performance differences between the two SOA conditions should decrease in Experiment 2. However, an alternative assumption is that rather than the TRT, the respective distribution of the SOAs influences whether the participants prefer a parallel or serial processing strategy. If this were the case, the SF condition should again rely on a mostly parallel processing strategy. In Experiment 2, we replicated the first experiment with SOAs 300 ms, 500 ms, and 1000 ms. Apart from this change, the experiment was identical to that of Experiment 1.

### Method

#### Participants

Forty-four students of the University of Cologne (5 men, mean age *M* = 21.56 years, SD = 2.57) participated in this experiment for exchange of either course credit or a payment of 3€. They were randomly assigned to the SF or LF condition (*N* = 23 in the SF and *N* = 21 in the LF condition). Due to a technical problem with data recording, two participants of the SF condition and one participant of the LF condition were excluded.

Apart from the prolonged SOAs, the stimuli and the procedure were as described in the "General method".

### Results and discussion

Again, the error rates were rather low and were unaffected by SOA or SOA condition (see Table 1; *F*s ≤ 1.41, *p*s ≥ 0.242 and *F*s ≤ 1.41, *p*s ≥ 0.242, for Task 1 and Task 2, respectively).

Figure 3a and 3b depict the mean RTs for Tasks 1 and 2 in the two SOA conditions. The 2 × 3 ANOVA with RT1 as dependent variable revealed a significant main effect of SOA (*F*(2,78) = 5.22, *p* = 0.020, *η*_*p*_^2^ = 0.12; *ε* = 0.626). Contrary to Experiment 1, the main effect of SOA condition was now significant (*F*(1,39) = 8.28, *p* = 0.006, *η*_*p*_^2^ = 0.18), indicating that the participants responded slower in the SF condition than in the LF condition. In addition, also the SOA condition × SOA interaction was significant (*F*(2,78) = 7.37, *p* = 0.006, *η*_*p*_^2^ = 0.16; *ε* = 0.626). As can be seen from Fig. 3a, this interaction was again caused by an increase of RT1 with increasing SOAs in the SF condition and the reversed pattern in the LF condition. Thus, contrary to the expectation derived from the TRT account, prolonging the SOAs did not reduce the RT differences between the two conditions in Task 1.

For RT2, the 2 × 3 ANOVA showed only a significant main effect of SOA (*F*(2,78) = 153.50, *p* < 0.001, *η*_*p*_^2^ = 0.79; *ε* = 0.768), reflecting the PRP effect. Neither the main effect of SOA condition (*F*(1,39) = 1.09, *p* = 0.303, *η*_*p*_^2^ = 0.03), nor the SOA condition × SOA interaction reached significance (*F*(2,78) = 1.01, *p* = 0.369, *η*_*p*_^2^ = 0.03). Despite the longer RTs in Task 1, participants in the SF condition showed no benefit in Task 2. This finding does not mirror the results obtained in Experiment 1, and fits neither with a mostly parallel nor a mostly serial processing strategy adopted in the SF condition. If the prolonged RT1 in the SF condition reflected a parallel processing mode, we should have also found at least a small beneficial effect in Task 2. Therefore, it seems probable that the slower processing of Task 1 in the SF condition might have been caused by other reasons than parallel processing. We will refer back to this in the “General discussion”.

## Experiment 3

In Experiment 3, we tested whether interspersing trials that required no Task 2 response would increase serial processing irrespectively of the SOA distributions. Such a manipulation should enhance the prioritization of Task 1 by preventing concurrent Task 2 processing (Koch et al., 2018; Mittelstädt & Miller, 2017). Specifically, we replicated Experiment 1, and included two additional stimuli in Task 1 (a lemon and grapes) which reliably announced the no-go trial in Task 2. Furthermore, the no-go Stimulus of Task 2 (a cat) was never associated with any response beforehand. Nevertheless, since such no-go trials could have produced a no-go BCE (Janczyk et al., 2014), we excluded these trials from the data-analyses of Experiment 3.

### Method

#### Participants

Forty students of the University of Cologne (8 men; mean age *M* = 23.58 years, SD = 3.00) participated in the experiment either in exchange for course credit or a payment of 3€. 19 participants were assigned to the SF, and 21 participants to the LF condition.

Apart from the exception that Task 1 contained two additional images, one of a yellow lemon and one of blue grapes, the procedure was identical to Experiment 1. These two stimuli led to the same yellow–blue responses but signaled participants that Task 2 will not require any response. There were 82.5% go trials and 17.5% no-go trials. The stimulus of Task 2 in these trials was always the image of a cat which was never assigned to any response key.

### Results and discussion

We first analyzed participants’ error rates. The mean error rates are presented in Table 1. Again, neither SOA nor SOA condition affected the mean error rates (see Table 1; *F*s ≤ 1.87, *p*s ≥ 0.179 and *F*s ≤ 1.14, *p*s ≥ 0.325 in Task 1 and Task 2, respectively).

Figure 3a and b show the mean RTs for Tasks 1 and 2 in the two conditions. For means of comparison between experiments, we did not include the Task 1 trials which were followed by a no-go trial in Task 2 because they might have produced a BCE. This seems also warranted because there was no significant difference between go and no-go trials and also no interaction between this and any other factor. The 2 (SOA condition) × 3 (SOA) ANOVA with RT1 as dependent variable showed a significant main effect of SOA (*F*(2, 76) = 6.04, *p* = 0.010, *η*_*p*_^2^ = 0.14, ε = 0.688) and of SOA condition (*F*(1,38) = 6.52, *p* = 0.014, *η*_*p*_^2^ = 0.15). In addition, also the interaction between SOA and SOA condition was significant, (*F*(2, 38) = 7.01, *p* = 0.006, *η*_*p*_^2^ = 0.16, *ε* = 0.688). Thus, the results of Experiment 3 resembled those of the former two experiments with the exception that, here, RT1 did not increase as a function of SOA lengths in the SF condition. Instead, the SOA condition × SOA interaction was solely caused by the LF condition which shows a shorter RT1 with longer SOAs.

With RT2 as dependent variable, the 2 × 3 ANOVA yielded only a significant main effect of SOA, (*F*(2, 76) = 199.58, *p* < 0.001, *η*_*p*_^2^ = 0.84, *ε* = 0.817). The main effect of SOA condition was not significant, *F*(1, 38) = 0.24, *p* = 0.625, *η*_*p*_^2^ = 0.01. The interaction just failed to reach the level of significance, *F*(2, 76) = 2.84, *p* = 0.064, *η*_*p*_^2^ = 0.07.

Thus, the longer RT1 in the SF condition suggest that participants were more likely to conduct the two tasks in parallel. As in Experiment 2, however, RT2 as a function of SOA did not differ significantly between the two conditions.

## Interim summary of the empirical findings

The empirical findings of Experiment 1 are in line with the TRT framework of Miller et al. (2009) that blocks with mostly short SOAs increase the likelihood of parallel processing of the two tasks, because parallel processing is, in this case, the more efficient strategy. We found the interaction between SOA condition and SOA for Task 1, and, in Task 2, the flatter slopes in the SF condition compared to the LF condition. However, even though the manipulations of prolonging the shortest SOA (Experiment 2) or enhancing the prioritization of Task 1 (Experiment 3) were thought to reduce the likelihood of parallel processing, the interactions between SOA condition and SOA in Task 1 remained significant in Experiments 2 and 3. Only the pattern of results of Task 2 was in line with the expectation that these manipulations reduced the probability of parallel processing. The patterns of Task 1 obtained in Experiments 2 and 3 are unexpected. As argued above, increasing the likelihood of serial processing should have speeded up RT1, thereby attenuating the differences between the SF and the LF conditions.

In addition, one further point in the data is noteworthy: When taking a closer look at RT1, it becomes obvious that the longer the SOAs, the slower the participants in the SF condition responded (at least in Experiments 1 and 2). This is what Miller et al. (2009; Fig. 3) had found as well. However, this result is contrary to what one would expect from the perspective of models assuming the possibility of parallel processing: With short SOAs, the overlap between the two tasks is larger than at long SOAs and, thus, response speed should increase from short to long SOAs. Therefore, this finding does not match the conclusion of Miller et al. (2009) suggesting that participants try to optimize the TRT by either preferring a mostly parallel or a mostly serial processing strategy.

## Results from the drift–diffusion model

The MCMC chains converged according to visual inspection of the traces (see Figs. 6, 7, 8 in the "Appendix") and the Gelman Rubin statistics (*Ȓ* were all close to 1). Also, the fit between the model and the empirical data was quite well. It was slightly better for Task 1 than for Task 2. Furthermore, since the error rate was rather low, the RT distribution of the erroneous responses was predicted less well than the RT distribution of the correct responses. The detailed model fit is shown in "Appendix".

### Parameter pattern for the drift rate *v*

**Fig. 4:**
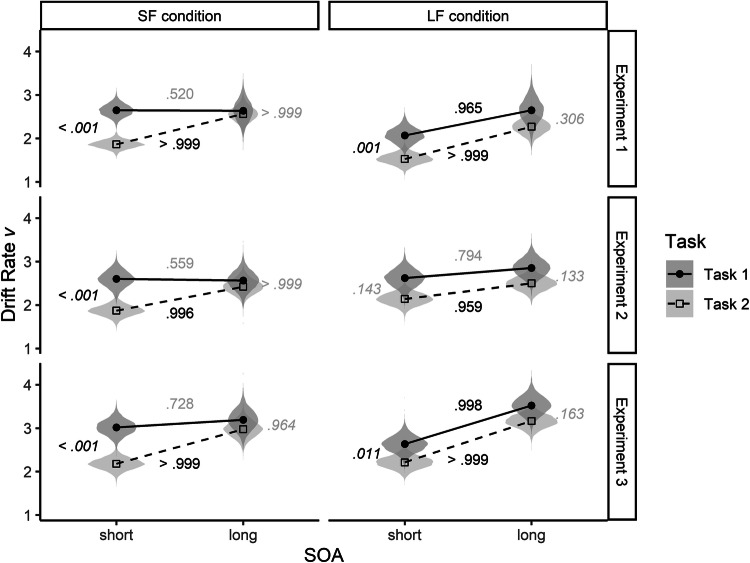
Posterior distribution of drift rate parameter estimates (violins) and mean of the distributions (points) of the SF and the LF conditions in Tasks 1 and 2 in Experiments 1–3. For better identification of statistically substantial differences, frequentist *p *values (italics) and Bayesian *P* values (non-italics) are displayed. The black font indicates *p* < 0.05 and *P* > 0.95, and the gray font any other values

As described in Fig. 2, we expected mostly parallel processing in the SF condition to lead to lower drift rates for short than for long SOAs in both tasks, reflecting the sharing of central capacities of parallel processing. By contrast, if the frequent long SOAs in the LF condition had biased the participants towards a mostly serial processing mode, the drift rate should be unaffected. Figure 4 depicts the results of the drift rate *v* as a function of Task, SOA, and SOA condition separately for the three experiments.

As can be seen from Fig. 4, we obtained rather similar results across the three experiments. However, they are not entirely in line with our expectations. In the SF conditions, the hypothesized increase in the drift rate from short to long SOAs appeared only for Task 2,* P* > 0.999 (Experiment 1), *P* = 0.996 (Experiment 2), and *P* > 0.999 (Experiment 3).^2^ By contrast, Task 1 drift rate was unaffected by SOA (all *P*s > 0.73).

In addition, in all three experiments, the drift rate for short SOAs in the SF condition was significantly higher in Task 1 than in Task 2, *t*(20) = 6.83, *p* < 0.001 (Experiment 1), *t*(20) = 5.78, *p* < 0.001 (Experiment 2), and *t*(18) = 8.66, *p* < 0.001 (Experiment 3). Thus, it seems as if participants allocated more of their resources to the processing of Task 1 than of Task 2 (Mittelstaedt & Miller, 2017) rather than processing both tasks fully in parallel and with equal importance.

For the LF condition, the results are surprising as well. Except for Experiment 2, the drift rates of both tasks increased significantly from short to long SOAs, *P* = 0.965 and *P* > 0.999 (Tasks 1 and 2 in Experiment 1), *P* = 0.998 and *P* > 0.999 (Tasks 1 and 2 in Experiment 3). Only in Experiment 2, in which we prolonged the shortest SOA, this increase in the drift rate disappeared for Task 1, but still was significant for Task 2, *P* = 0.959. Thus, the drift rates do not fit our expectation of a mostly serial task processing in the LF condition (see Fig. 2).

In addition, when comparing the drift rate between the two experimental conditions, the drift rate of Task 1 with short SOAs was not lower in the SF condition. In Experiment 1, which was mostly a replication of the Miller et al. (2009) experiments, the drift rates for both tasks were even higher in the SF than in the LF condition, *t*(34.42) = 3.27, *p* = 0.002 (Task 1 in Experiment 1), and *t*(33.76) = 3.04, *p* = 0.005 (Task 2 in Experiment 1). Once again, this pattern of results seems to be at odds with the assumption of mostly parallel processing when short SOAs were frequent.

### Parameter pattern for non-decision time *t*_0_

**Fig. 5:**
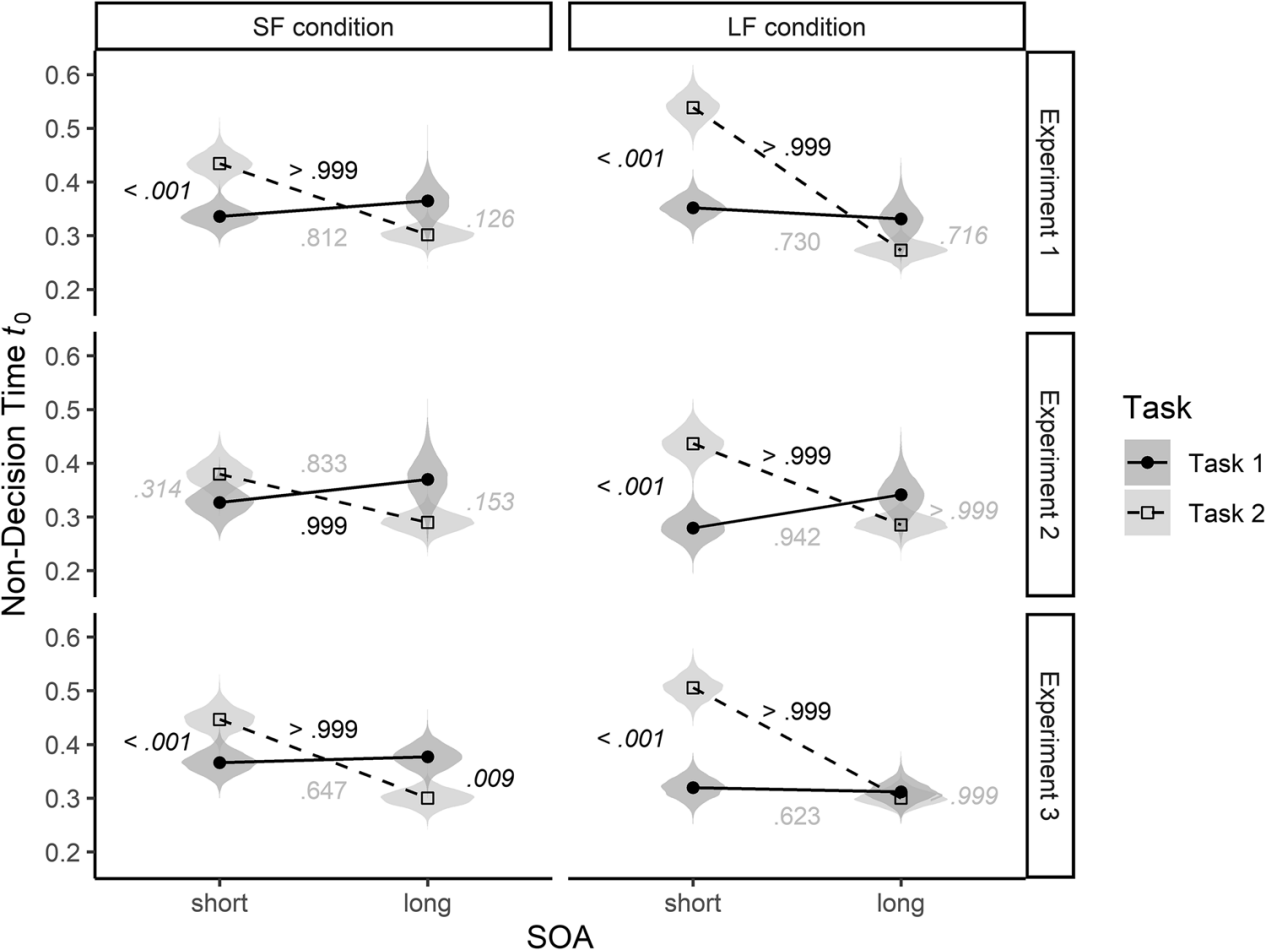
Posterior distribution of non-decision time parameter estimates (violins) and mean of the distributions (points) of the SF and the LF conditions in Task 1 and 2 in Experiments 1–3. For better identification of statistically substantial differences, frequentist *p *values (italics) and Bayesian *P* values (non-italics) are displayed. The font is black for *p* < 0.05 and *P* > 0.95, and gray of any other values

**Fig. 6:**
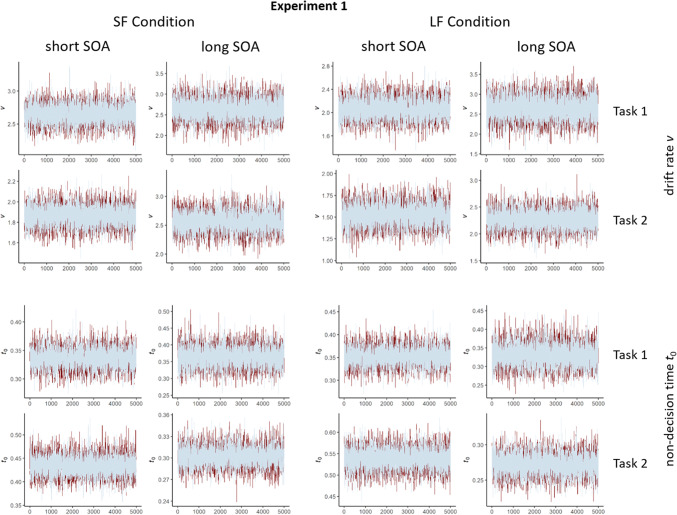
Trace plot for the drift rate *v* and the non-decision time *t*_0_ in both SOA conditions of Experiment 1. The plots are separated by SOA (short vs. long) and Task. The five independent chains sample from the same posterior distribution of the parameter

**Fig. 7:**
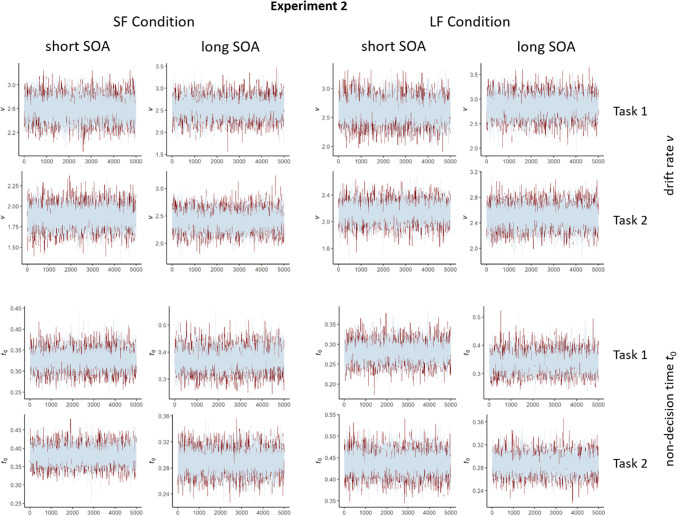
Trace plot for the drift rate *v* and the non-decision time *t*_0_ in both SOA conditions of Experiment 2. The plots are separated by SOA (short vs. long) and Task. The five independent chains sample from the same posterior distribution of the parameter

**Fig. 8:**
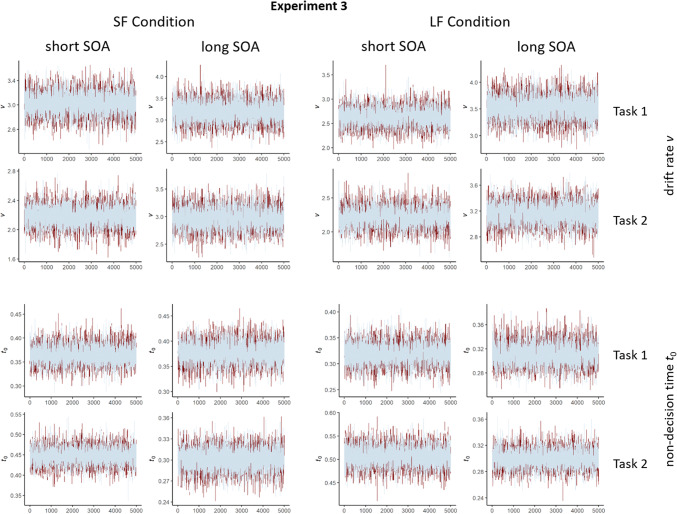
Trace plot for the drift rate *v* and the non-decision time *t*_0_ in both SOA conditions of Experiment 3. The plots are separated by SOA (short vs. long) and Task. The five independent chains sample from the same posterior distribution of the parameter

According to our hypotheses, the non-decision time *t*_0_ should not differ between short and long SOAs or between Task 1 and Task 2 in the SF condition. By contrast, participants in the LF condition should show longer non-decision times in Task 2 with short than with long SOAs, and additionally longer non-decision times for Task 2 than for Task 1 with short SOAs (see Fig. 2). Figure 5 depicts the non-decision time *t*_0_ in Tasks 1 and 2 as a function of SOA, SOA condition and Experiment.

Again, contrary to our expectations, we found strong indicators for serial processing in the SF condition. In particular, in Task 2, the non-decision time was larger with short SOAs compared to long SOAs, all *P*s > 0.995. Also, with short SOAs, the non-decision time was larger in Task 2 compared to Task 1 in Experiments 1 and 3 (*t*(20) = 5.97, *p* < 0.001 and *t*(18) = 6.32, *p* < 0.001, in Experiments 1 and 3, respectively).

For the LF condition, the pattern of results was as predicted. In all three experiments, the non-decision time was larger in Task 2 with short SOAs than with long SOAs, all *P*s > 0.999. Furthermore, and also as expected, for short SOAs, the non-decision time in Task 2 was larger than in Task 1 in all three experiments, *t*(15) = 8.71, *p* < 0.001 (Experiment 1), *t*(19) = 6.29, *p* < 0.001 (Experiment 2), and *t*(20) = 10.75, *p* < 0.001 (Experiment 3).

Thus, in both SOA conditions, the non-decision time was elevated in Task 2 with short SOAs, likely reflecting serial processing. Importantly, comparing these differences between Task 2 with short SOAs versus with long SOAs between conditions revealed that they were substantially smaller in the SF than in the LF condition. To compare the two SOA conditions, we subtracted the posterior distribution of Task 2 with long SOAs from the posterior distribution of Task 2 with short SOAs. We then compared the resulting distribution of differences between the SF and LF conditions. The difference in the non-decision time between Task 2 with short SOAs and Task 2 with long SOAs was smaller in the SF condition compared to the LF condition, *P* = 0.999 (Experiment 1), *P* = 0.937 (Experiment 2, marginal), *P* = 0.953 (Experiment 3). Likewise, the differences between Task 2 and Task 1 with short SOAs were also smaller for the SF than for the LF condition, *t*(30.66) = 3.12, *p* = 0.004 (Experiment 1), *t*(38.76) = 2.83, *p* = 0.007 (Experiment 2), *t*(36.87) = 4.50, *p* < 0.001 (Experiment 3).

Taken together, the analysis of the non-decision time suggests that despite having induced a parallel processing strategy by presenting frequently short SOAs (Miller et al., 2009), we still found a rather strong bias towards serial processing in all three experiments, albeit to a smaller extent in the SF condition.

## General discussion

In the first part, we reported three dual-task experiments investigating the conclusion of Miller et al. (2009) that participants adapt their processing strategies to the given distribution of SOAs with the goal of minimizing the TRTs. For this purpose, we realized two experimental conditions: the SF condition with mostly short SOAs and the LF condition with mostly long SOAs. The participants should prefer a parallel processing mode in the SF condition because in this case, this strategy should lead to shorter TRTs. Contrary, a serial processing mode should be more efficient when long SOAs are most frequent, as was the case in the LF condition. In the second part of the article, we used the drift–diffusion model approach (Ratcliff & McKoon, 2008) to compare performance in the two conditions in more detail. In terms of this approach, we expected that the preference of the parallel processing mode in the SF condition would be reflected primarily in a reduced drift rate at short SOAs; whereas, the mostly serial processing strategy in the SF condition would influence the non-decision time at short SOAs. While we obtained rather consistent findings across the three experiments, they do not entirely fit the predictions derived from Miller et al.’s TRT framework and, thus, require further discussion.

First, the RT pattern of Experiment 1 replicated the main findings of Miller et al. (2009). Comparing the two SOA conditions revealed that participants in the SF condition were slower in Task 1 and slightly faster in Task 2. At first glance, this is what would be expected under the assumption of models assuming the possibility of parallel task processing. With short SOAs, parallel task processing should lead to a long RT1 and a short RT2. Although we found this interaction, our findings revealed that in the SF condition, RT1 increased with longer SOAs. As already mentioned, this mirrors the findings of Miller et al. (2009) and, thus, is not unique to our current results (see Experiments 1 and 2 of Miller et. al, 2009; see also, Logan & Gordon, 2001; Experiment 3; Mittelstaedt & Miller, 2017). In addition, in Experiment 2, as we prolonged the shortest SOA from 100 to 300 ms to foster serial processing in the SF condition, we found almost the same RT1 increase in the SF condition. If participants had tried to optimize the TRTs by adopting a mostly serial processing strategy (Miller et al., 2009), this RT1 increase should have disappeared. This was only the case in Experiment 3, when we introduced a few Task 2 no-go trials to increase prioritization of Task 1. Thus, overall, these findings suggest that the longer RT1 in the SF condition in comparison to the LF condition is ambiguous in supporting the assumptions of the capacity-sharing account. Only the findings concerning Task 2 hint to mostly parallel processing in this condition by showing the flatter slopes in Experiment 1. This difference between the two SOA conditions disappeared, as expected, in Experiments 2 and 3.

Second, the results from the drift–diffusion model analysis also deviated from the predictions derived from the TRT framework. The drift rate does not seem to support the assumption of mostly parallel processing in the SF condition. In all three experiments, we only found the expected increase in the drift rate for Task 2, while Task 1 was almost entirely unaffected by SOA. Furthermore, in Experiment 1, the drift rate for both tasks was significantly higher with short SOA in the SF than in the LF conditions. Sharing capacities between tasks should have decreased the drift rate in the SF condition, in particular with short SOAs in Experiment 1. In addition, we expected that the non-decision time would decrease from short to long SOAs only in Task 2 within the LF condition. However, we found this decrease also in the SF condition, though to a significantly lesser extent. Thus, our results from the drift–diffusion model do not unambiguously support the assumption that participants had adapted their processing modes to optimize the TRTs, either.

One possibility to cope with these rather puzzling findings is to assume that the frequency manipulation of the SOAs has not primarily induced a parallel versus serial processing mode. An alternative assumption could be that presenting mostly short SOAs might have led to grouping of the responses to the two tasks (cf. Lien, Schweickert, & Proctor, 2003; Pashler, 1994a; Pashler & Johnston, 1989). For two reasons, however, the assumption of response grouping is unlikely in the present experiments. First, if participants withheld the first response until the second response had been selected, the non-decision time of Task 1 should have increased with SOA. However, in none of the experiments, we found such an increase. Second, even though the amount of IRIs smaller than 100 ms was higher in the SF than in the LF condition, it decreased in all experiments from approximately 20% with short SOAs to approximately 5% with long SOAs in the SF condition. Response grouping should have led to a rather stable amount of short IRIs (for similar arguments, see Miller et al., 2009). Furthermore, excluding these short IRIs did not alter the qualitative pattern of results.

As a second alternative, it is conceivable that the frequency manipulation in our experiments affected higher-order (executive) control mechanisms as is assumed in cognitive control models (cf. Fischer & Plessow, 2015; Lehle & Hübner, 2009; Logan & Gordon, 2001). For instance Lien, Ruthruff, Cornett, Goodin, & Allen (2008), provided evidence that even when the secondary task is presented in advance to the primary task in a PRP paradigm (i.e., with a negative SOA), participants maintain the order of task processing. This suggests that participants might have represented a specific task order that guided their dual-task performance (see Luria & Meiran, 2003, for similar assumptions). With regard to our experiments, one possibility is that the distribution of SOAs influenced the temporal expectancies of participants which, in turn, might have helped them to schedule task processing (Bausenhart, Rolke, Hackley, & Ulrich, 2006; Lohmann, Herbort, Wagener, & Kiesel, 2009; Los & Horoufchin, 2011).

According to the body of research on time expectancy, humans seem to represent time intervals not in an absolute but in a relative manner (Bausenhart, Bratzke, & Ulrich, 2016; Bratzke & Bryce, 2016; Los, Kruijne, & Meeter, 2017). Time expectancies are used to prepare for an upcoming task. Even if the specific response is unknown yet, a decisive process in response preparation may be a “time-based general preparation” process (Langner, Steinborn, Eickhoff, & Huestegge, 2018, p. 1331).

Transferring the notion of time expectancy to the current study, one prediction is that participants would show an advantage in trials containing the most frequent SOA. Accordingly, participants in the SF condition should be faster when SOAs are short and slower when they are long. The LF condition should show the reverse pattern.

For Task 1, the RT pattern of both conditions fits these hypotheses almost perfectly: RTs were longer when presenting the less likely SOA.^3^ Only in the SF condition of Experiment 3, this increase of RTs from short to long SOA is missing. A plausible explanation is that the few interspersed no-go trials in the secondary task might have prompted participants to adapt their processing strategy in a trial-by-trial manner (Mittelstaedt & Miller, 2017). Two findings from the drift–diffusion model further fit well with the time-expectancy notion. First, the drift rate for Task 1 in the SF condition was relatively high. Second, the drift rate in the LF condition increased from short to long SOAs. Against the backdrop of time expectancy, this can be explained by assuming that participants in the LF condition were simply less prepared at unexpected short SOAs leading to the lower drift rate (Langner et al., 2018). However, this increase of the drift rate was not found for Experiment 2. Here, the prolongation of the shortest SOA from 100 to 300 ms might have been sufficient to compensate the initial lack of task preparation at short SOAs. This argument is in line with findings in the field of time expectancy: In experiments in which the foreperiods (i.e., the temporal distance between warning signal and reaction stimulus) were manipulated, a general preparedness advantage for long foreperiods due to sufficient preparation time was found, despite worse temporal estimation with longer foreperiods (De Jong, 1995; Langner et al., 2018; Niemi & Näätänen, 1981). Presupposing that in our experiments, the Task 2 stimulus functions as a warning signal, the overall result pattern for Task 1 is well in line with the assumption that participants relied on temporal expectancies established by the manipulation of SOA frequencies. In addition, this account can explain why participants in the SF condition showed the increase in RT1s for longer SOAs.

For Task 2, the picture of results is less clear. In line with the time-expectancy account, the behavioral data show that participants in the SF condition were, at least in Experiment 1, faster at short SOAs than the LF condition. Likely, they were simply better prepared for this frequent SOA. Yet, the reverse benefit for long SOAs is missing in the LF condition. Again, this can be explained by assuming that long SOAs generally led to a better performance, as these trials provide sufficient time to prepare for an upcoming task even when participants were less prepared for these unexpected long SOAs (De Jong, 1995; Langner et al., 2018). Thus, with this additional assumption, the behavioral data of Task 2 can be explained in the context of a time expectancy account as well.

However, the Task 2 findings are ambiguous in supporting the time expectancy account because the capacity-sharing account of Miller et al. (2009) would come to rather the same predictions. The results from the drift–diffusion model might help clarifying this ambiguity. The findings for Task 2 suggest that, according to our prediction of longer non-decision times with shorter SOAs, participants not only in the LF, but also in the SF condition relied on a mostly serial processing strategy. In both conditions, the non-decision time in Task 2 decreased with longer SOA. Concurrently, in the SF condition, the drift rate in Task 2 was lower than in Task 1 with short SOAs and increased from short to long SOAs. These latter findings can be reconciled with the assumption of, for instance, the capacity-sharing account only if one assumes that participants strongly prioritized Task 1 processing (Tombu & Jolicoeur, 2003). Task 2 processing then progresses rather slowly unless Task 1 has been finished. In some cases, this strong prioritization of Task 1 might have led to serial processing reflected by the longer non-decision times for Task 2 when the SOA was short.

Taken together, while the data pattern of Task 1 seems to be better explained by focusing on higher-order control processes like the role of time expectancy, the findings for Task 2 are in line with both the time expectancy and the capacity-sharing accounts of Miller et al. (2009).

### Why might time expectancy play a role in dual-tasking?

It is conceivable that expecting a short (SF) or long (LF) SOA can involve a temporal structuring of the task material that extends beyond the current trial. Based on the literature on time-expectancy, we suggest that participants might interpret the appearance of the second stimulus after varying SOAs as an externally presented rhythm (Adams & Creamer, 1962; Grosjean, Rosenbaum, & Elsinger, 2001; Wing & Kristofferson, 1973a, b). We further assume that they try to adopt a global rhythm holding for most of the trials within a block. As shown by, for instance, Krampe, Mayr and Kliegl (2005), switching between different rhythms requires mental effort. Therefore, a less effortful strategy might be to adopt the global rhythm that is comfortable for most of the presented SOAs (for an analogous point, see Lehle & Huebner, 2009).

In the current experiments, the different SOA distributions in the two experimental conditions led to the experience of different external rhythms: a relatively fast one in the SF condition, and a rather slow one in the LF condition. In the SF condition with the rather fast rhythm, participants expect the secondary stimulus to occur immediately after the primary stimulus. They might have used this rhythm of the appearance of the secondary task as an external impulse generator to start processing the primary task. In the case of unexpected long SOAs, the later appearance of the secondary task leads to deferred processing of the primary task because participants must have realized that they had to initiate Task 1 processing without this external impulse generator. This then might have caused the rather slow responses for long SOAs in the SF condition. In the LF condition, by contrast, the slower rhythm and, thus, the expectancy of longer time intervals might have hindered participants to use the appearance of the secondary task as an external impulse generator. Instead, they immediately started to process the primary task after the primary stimulus was presented. In case of a rare short SOA, they had to reorganize their global scheduling strategy, thus needing more time to respond to the stimulus.

Thus, unlike Miller et al. (2009), our notion of time expectancy does not focus primarily on the distinction between parallel versus serial task processing. Rather, we suspect that, in a similar vein as with other parameters in cognitive control models, the different frequencies of the SOAs might have biased participants to adopt a global temporal expectancy when to process the secondary stimulus. Temporal expectancy might either influence when the stimulus is being processed by setting up when to attend the stimulus. As such, this would not be specific to dual-tasking. Alternatively, this temporal expectancy might help to more optimally set task control parameters (e.g., attentional breath, stimulus prioritization; e.g., Logan & Gordon, 2001). This might enable participants to optimally schedule the dual-task processing (cf. Schubert et al., 2017) based on the respective most frequent SOA without the need for choosing either a serial or parallel processing strategy.

There is at least one (not mutually exclusive) alternative explanation proposed by Strobach, Salminen, Karbach, & Schubert (2014). These authors assume that participants can instantiate either both tasks together at the beginning of each trial or separately, immediately before processing the respective task. By instantiation, Strobach et al. (2014) mean the process of loading task rules into working memory. According to this assumption, the high proportion of short SOAs in the SF condition could have led participants to instantiate both tasks together at the beginning of each trial. When the SOA is short, Task 2 processing should be faster than in the LF condition because with frequently long SOAs, participants must go back to activating the secondary task before it can be processed. This additional switching component then prolongs RT2 in the LF condition. The longer the SOAs, the smaller is the effect of this extra loading time on RT2. Thus, according to this proposal, the manipulation of SOA distributions does not affect a parallel or serial processing mode either. Rather, it affects the task coordination processes (Strobach & Schubert, 2017).

### Limitations of the current study

Two limitations of the current study should be noted: First, the shortest SOA in our experiments was 100 ms (80% of the trials in the SF condition). This SOA is slightly longer than Miller et al.’s (2009) SOA of 16 ms (40% of the trials in Experiment 1), but it is faster than the SOA of 133 ms which they presented in 30% of the trials. Thus, it is not that likely that the longer SOA in our study might have altered the results. Furthermore, our findings of Experiment 1 replicated those of Miller et al. (2009). Nevertheless, it could be worthwhile to replicate exactly the SOAs of Miller et al. and analyze these findings with the drift–diffusion model. This should involve exploring the potential effects of administering the SOA frequency manipulation within subjects (Miller et al., 2009) or between subjects (current study). In the between-subjects variant, it is not an issue how quickly and to what extent participants adopt to a changed SOA distribution. Furthermore, post-experimental questioning to probe into awareness could be included. Yet, the within-subjects variant is beneficial in terms of statistical power.

Although there are multiple studies which have validated the diffusion model (e.g., Durst & Janczyk, 2019; Lerche & Voss, 2017), some studies have warned to map the diffusion model parameters exclusively to a single cognitive process (e.g., Rieger & Miller, 2019). Accordingly, the results of the diffusion model analyses should be interpreted cautiously. We believe, however, that combining conventional RT analyses (which must be interpreted similarly cautiously due to the many processes that are captured in the RTs) and diffusion model analyses helps to obtain a more complete picture of the topic under investigation.

A further issue concerns our experimental tasks. In all three experiments, the two tasks were always visual–manual tasks which might have increased the interference between them. This might have hindered or reduced the use of a parallel processing mode (cf. Fischer, et al., 2014; Fischer & Plessow, 2015). The findings of Miller et al. (2009; Experiment 3) suggest that presenting two tasks in different modalities slightly increases parallel processing in the SF condition. Thus, it is possible that in our study using the same modality in both tasks could have reduced the likelihood of a parallel processing of both tasks.

## Conclusion

We started our series of experiments with the intention to provide further evidence for parallel and serial task processing. The results, however, leave us at a point where we tend to argue that the RT pattern and the results of the drift–diffusion model analysis might not reflect parallel versus serial processing, but rather simply an efficient strategy based on participants’ time expectancies referring to the occurrence of the secondary task stimulus. Such a strategy might enable participants to reduce their mental effort while processing the dual task. Whether this might be true for parallel and serial processing in general remains an open question for further research.

## Figures and Tables

**Fig. 9:**
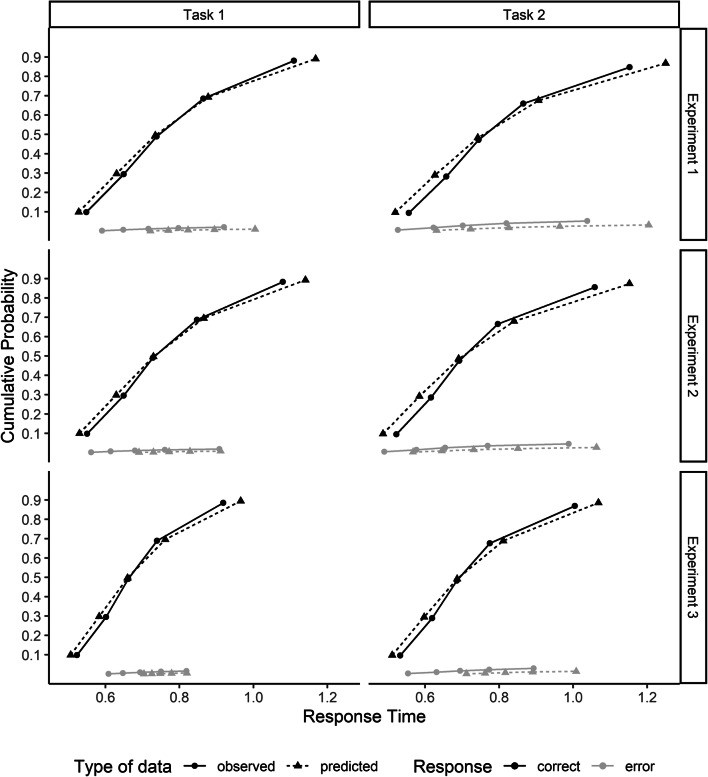
Cumulative probability plot for experiments 1–3 and Tasks 1 and 2 based on group-level parameter estimations

**Fig. 10:**
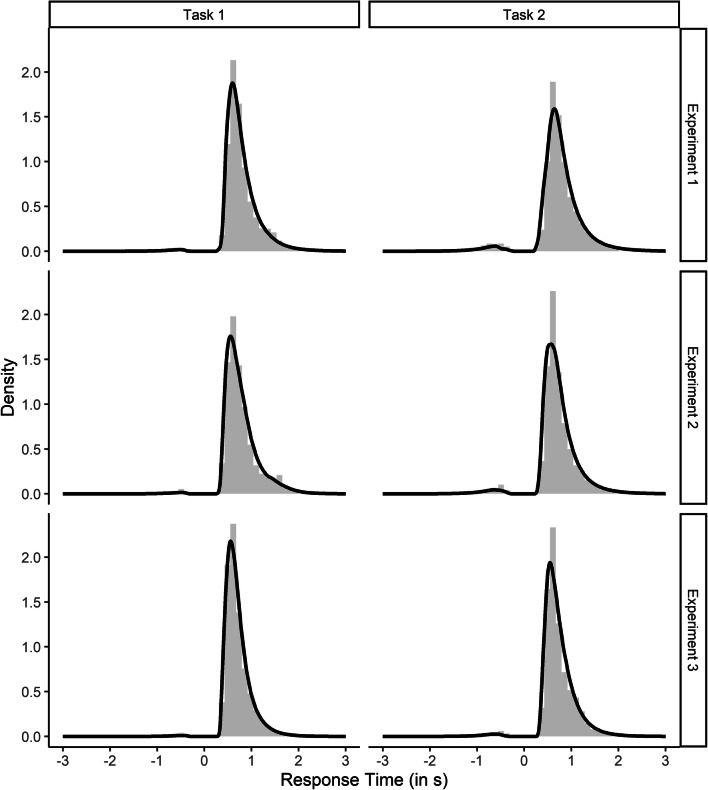
Observed response time distribution and density function of the response time distribution predicted by the diffusion model based on the group-level parameter estimates. Positive response times refer to correct responses, negative response times to erroneous responses

## A1: MCMC convergence

To evaluate model fit, we visually inspected the trace plots of the group-level parameter estimates. The absence of a trend in the trace indicates convergence (Hamra, MacLehose, & Richardson, 2013). Figures 6, 7, 8 show the trace plot of the traces for the drift rate *v* and the non-decision time *t*_0_ in Tasks 1 and 2 of the three experiments for five independent chains, each. In all experiments, there was clearly no trend in the traces and on each iteration, a parameter estimate was randomly sampled from the posterior distribution, indicating convergence. Furthermore, the five independent chains all sampled from the same posterior distribution of the respective task and experiment. In short, the trace plots indicated convergence for all parameters in both tasks of the three experiments.

Another method to evaluate convergence is the Gelman Rubin statistic *Ȓ* (Gelman & Rubin, 1992). This statistic compares the variability within a posterior distribution with the variability between a number of independently sampled posterior distributions of a parameter estimate. Values near 1 indicate convergence. *Ȓ* should not exceed 1.02 (Wiecki et al., 2013). We ran our models five times and computed *Ȓ* based on these five chains for each diffusion model parameter in each condition for each participant and on the group level. All *Ȓ* were close to 1 and none exceeded 1.02, indicating chain convergence in all parameters and all experiments. Thus, the posterior distributions of all nodes were reliably estimated.

Overall, the results of the visual inspection of the trace plots and the Gelman Rubin statistic consistently indicate convergence of the MCMC chains. Accordingly, the parameter estimates can be reliably reproduced which is the basis for interpreting the parameters.

## A2: Model fit

To assess the model fit, we conducted posterior predictive checks. We randomly drew a set of parameters from the posterior distribution and generated RTs and response accuracy according to the diffusion model based on this parameter configuration. We repeated this procedure 500 times, resulting in 500 sets of RT and accuracy data which served as the basis to compare the generated data based on the parameter estimates to the observed data.

For each generated data set, we computed quantiles of the RTs for correct and incorrect responses, resulting in a distribution of the quantiles. We then compared the mean of these distributions to the corresponding quantiles of the observed RTs (Figs. 9, 10).

In all three experiments, the predicted RT quantiles for correct responses were close to the observed quantiles (Fig. 9). In general, the fit for Task 1 seemed to be slightly better than the fit for Task 2. For Task 2, the model produced slightly slower RTs at the tail of the distribution compared to the observed RTs (see also Fig. 10). The head of the distribution yielded a good fit to the observed data in Task 2, too. The model also predicted the error rate well. However, given that the error rate was very low in all experiments and especially in Task 1 (see Table 1), the predicted response quantiles deviated from the observed quantiles.

## A3: Technical aspects of the estimation procedure

There are numerous procedures to estimate the parameters of the diffusion model. Conventional procedures provide point estimates of the parameters (e.g., maximum likelihood, chi-square approach; Voss, Voss, & Lerche, 2015), i.e., they retrieve the most likely value of the parameters. Bayesian approaches of parameter estimation, however, provide a posterior probability distribution of parameter estimates. While these distributions also contain information about the probability of parameter values, they additionally provide information about the certainty of parameter estimates (Wiecki et al., 2013). Wide distributions cover a large range of parameter values indicating uncertainty of the estimates, while narrow distributions only cover a small range of parameter values indicating high certainty of the estimates. Furthermore, unlike conventional procedures, hierarchical Bayesian approaches estimate the parameters of all participants on the individual level and on the group level simultaneously, taking into account similarities between the individuals. As a consequence, these approaches may provide more precise parameter estimates and may need less trials per person for the parameter estimation compared to conventional estimation procedures (for a comparison of the estimation procedures, see Wiecki et al., 2013).

During Bayesian parameter estimation, an algorithm draws samples of parameter estimates. This parameter sampling produces a trace of parameters. At the beginning of the estimation procedure, there is a trend in the trace, indicating that the algorithm is not yet sampling from the posterior distribution of the parameter. The samples of parameters which cover this trend need to be discarded because they are not part of the posterior distribution. This is why a burn-in period is defined specifying the number of initial draws which are discarded. To ensure that in the final parameter sample, parameters were only sampled from the posterior distribution, indicating convergence, the trace plots can be examined (Figs. 6, 7, 8). The trace plots show the value of a given parameter (vertical axis) for each draw from the posterior distribution (horizontal axis). At convergence, there should be no trend in the trace, i.e., the parameter values were randomly drawn from the posterior distribution. Furthermore, when estimating the same parameter multiple times and displaying the traces in the same trace plot, the traces should mix, as is the case for all trace plots in Figs. 6, 7, 8. This indicates that the algorithm has arrived at the same posterior distribution for all independent parameter estimations.

Note that hierarchical Bayesian parameter estimation as implemented in the HDDM Toolbox (Wiecki et al., 2013) is not restricted to the diffusion model. It is a global procedure which can be used to estimate parameters in a multitude of contexts (Kruschke, 2010; Lee & Wagenmakers, 2013).

## Footnotes

1 We are aware that a between-participants design is less powerful than a within-participants design. However, due to practical considerations, we chose the between-participants design. These considerations were partly due to the fact that we did not want our participants to endure twice the length of the experiment which would have been inevitable in a within-participants design. See also considerations in favor of a between-subjects approach in the “General discussion”.

2 A *P* value larger than 0.950 indicates significant effects.

3 One way to quantify the expectancy effects is to subtract the RT for the respective frequent SOA from the RT for the respective infrequent SOA condition (while ignoring the middle SOA) within the SF and the LF condition, and then averaging across the SF and LF conditions. Thereby, SOA effects average out and expectancy effects can be seen in isolation. For RT1, the expectancy effects were *M* = 143.26 ms, SD = 177.77 ms (Exp. 1), *M* = 73.61 ms, SD = 152.74 ms (Exp. 2), and *M* = 61.47 ms, SD = 134.58 ms (Exp. 3). For RT2, the expectancy effects were *M* = 68.91 ms, SD = 76.49 ms (Exp. 1), *M* = − 2.86 ms, SD = 111.86 ms (Exp. 2), and *M* = 35.03 ms, SD = 107.35 ms (Exp. 3).
